# The company Christmas party and employee happiness

**DOI:** 10.1038/s41598-023-27473-y

**Published:** 2023-01-07

**Authors:** Hannes Zacher

**Affiliations:** Wilhelm Wundt Institute of Psychology, Leipzig University, Neumarkt 9-19, 04109 Leipzig, Germany

**Keywords:** Psychology, Human behaviour

## Abstract

Many companies host an annual Christmas or holiday party which, for many employees, represents a fun, meaningful, and ritualized event closely linked to the organization’s culture. However, the factors that relate to employees’ satisfaction or dissatisfaction with this event, as well as to their positive and negative affect associated with this event, are currently not well understood. Accordingly, the current study aimed to explore how employee characteristics (e.g., demographics, employment characteristics, attitudes), organizational characteristics (i.e., human relations, open systems, rational goal, internal process culture), and event characteristics (e.g., location, activities, heavy drinking, inappropriate behavior of supervisors or colleagues, ritual features) are related to employee happiness. Data were provided by 359 employees from various organizations in Germany, who completed an online survey at the beginning of January 2019. Results showed that a human relations culture, an external location, fun activities, informality, and symbolism predicted higher employee satisfaction with the Christmas party. In contrast, heavy drinking and formality predicted higher dissatisfaction with the Christmas party, and longer organizational tenure, a human relations culture, a speech, providing both alcoholic and non-alcoholic beverages, and symbolism predicted lower dissatisfaction. Furthermore, employee age, organizational identification, involvement in planning, a human relations and an internal process culture, a speech, providing both alcoholic and non-alcoholic beverages, heavy drinking, supervisor and coworker inappropriate behavior, formality, and symbolism were differentially associated with high- and low-arousal positive and negative affect. These findings suggest several directions for future research on company parties and have practical implications for organizations and party planning committees.

## Introduction

Celebrations and parties are important physical manifestations or artifacts of an organization’s culture, which involves the shared assumptions, values, and beliefs of its members^1,2^. Many employers host an annual Christmas or holiday party, typically in December, and employees often eagerly anticipate this festive and social occasion. The company Christmas party is a relatively rare (i.e., annual), but particularly fun, meaningful, and ritualized event for many employees, that has been neglected in the broader literature on fun in the workplace^3–5^. Although a number of articles have considered the link between Christmas and organizations more generally^6,7^, including gift exchanges among business partners^8^ and the wearing of festive headgear at work^9^, there currently is very little empirical research on the company Christmas party. An exception is an early ethnographic study that analyzed the Christmas party of an advertising agency and characterized it as a “particularly dramatic occasion within the social process of the agency” [^10^, p. 463]. Although this qualitative study significantly advanced understanding of the meaning of the Christmas party for employees, quantitative research on employees’ happiness associated with this important organizational ritual is so far missing in the literature. This lack of quantitative studies prevents evidence-based recommendations for future research and practical recommendations for organizations.

**Figure 1:**
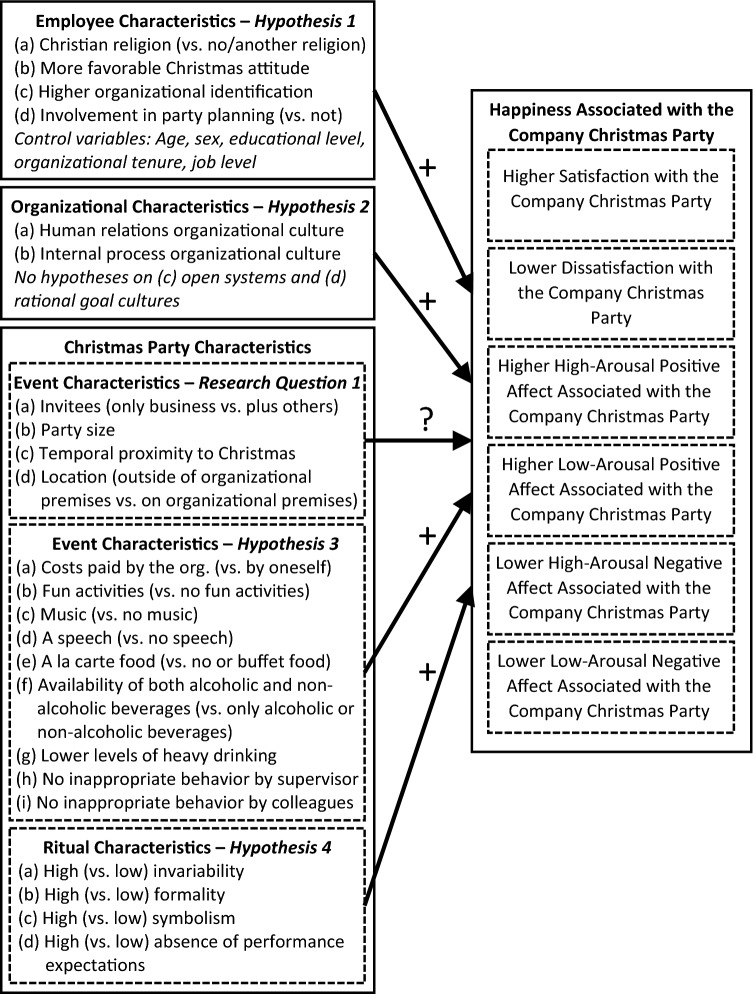
Conceptual model and hypotheses.

The overarching purpose of this article is to obtain a better understanding of the relative importance of various predictors of employee happiness associated with the company Christmas party. Thereby, this article aims to extend the broader literature on fun in the workplace^3,11^ by exploring potential antecedents of higher employee happiness associated with this special, and typically fun and meaningful, annual event. More specifically, the main goal of this study is to examine various employee, organizational, and event-related factors that may be related to employees’ satisfaction or dissatisfaction with the company Christmas party, as well as to their positive and negative affect associated with this event (see Fig. 1 for an overview of the conceptual model and hypotheses). Satisfaction and affect are considered important dimensions of people’s subjective well-being or happiness^12,13^. Whereas satisfaction and dissatisfaction describe the extent to which employees think that the Christmas party went well or not, respectively (i.e., because it met their expectations and needs, or not), positive and negative affect entail the frequency of people’s positive and negative emotions at the party, respectively. Consistent with research showing that positive and negative affect represent two distinct and relatively independent dimensions rather than opposite poles of a single continuum^14^, satisfaction and dissatisfaction are also conceptualized and measured here as two separate dimensions. The rationale for this distinction is that, on the one hand, low dissatisfaction with the company Christmas party is not equivalent to high satisfaction with the party. On the other hand, low satisfaction with the party does not mean that employees are highly dissatisfied with the party^15,16^. Researchers have further distinguished between high- and low arousal positive (e.g., excited vs. calm) and negative (e.g., stressed vs. sad) affect^17^.

As potential correlates of employee happiness, I explored various theoretically-derived employee characteristics (e.g., demographics, employment characteristics, attitudes), organizational characteristics (i.e., human relations, open systems, rational goal, internal process culture), and event characteristics (e.g., location, activities, heavy drinking, inappropriate behavior of supervisors or colleagues, ritual features). The distinction between employee, organizational, and event characteristics is based on affective events theory^18^ and conceptual work on fun in the workplace^3^, which distinguish these three broad categories to systematically investigate predictors of employee attitudes and well-being. To empirically examine my assumptions, I collected data from 359 employees from various organizations and occupations in Germany in January 2019 (i.e., more than one year before the onset of the COVID-19 pandemic in Germany, which prevented company parties^19^).

This study contributes to the literature in at least three important ways. First, I extend research on employee happiness^20,21^ by examining satisfaction and dissatisfaction as well as positive and negative affect associated with an important annual event in many organizations, the Christmas or holiday party. Satisfaction and affect are key components of happiness^22^, but it is currently unknown what factors are associated with these outcomes in the context of a company Christmas party. Second, I advance the emerging literature on fun in the workplace^3^, which so far has focused on everyday activities and experiences. In contrast, the company Christmas party as a relatively rare (i.e., annual), but typically fun, meaningful, and ritualized event for employees, has been neglected. Third, consistent with affective events theory^18^ and conceptual research on fun at work^3^, I categorize potential correlates of employee happiness associated with the company Christmas party into employee, organizational, and event characteristics. Although much research exists on individual difference predictors of employee happiness, such as demographics, appraisals, and coping strategies^19^, organizational and event characteristics have been neglected. Accordingly, I focus in the current study on four dimensions of organizational culture based on the well-established competing values theoretical framework^23^, as well as various specific characteristics of the Christmas party highlighted in previous research, such as location, fun activities, and alcohol consumption^10^. Moreover, I draw from the psychological literature on rituals^24^ to derive hypotheses on the role of ritual features (e.g., symbolism) for employee happiness associated with the Christmas party.

## Theoretical background and literature review

In the popular 2016 comedy movie *Office Christmas Party*, the local manager of an internet company needs to win over a wealthy client and, to this end, throws an exorbitant company Christmas party that involves heavy drinking and ends in chaos^25^. Numerous media reports have focused on reasons for and against the company Christmas party^26^, as well as (in-)appropriate employee behavior during the party^27^. With regard to the latter, two articles focused on legal employment relations issues regarding the company Christmas party. First, an article in a magazine by the American Bar Association (“Careful with that eggnog: Perils and pitfalls of the office holiday party”) concluded: “Despite the legal risks and etiquette minefields, holiday office parties are important tools for building and maintaining a positive working environment. With adequate planning and a focus on professionalism, they can be a positive experience for all” [^28^, p. 15]. Second, a “question and answers” article in *Employment Relations Today* suggested that, “The ‘most wonderful time of the year’ is often one of employers’—and employers’ lawyers’—least favorite, chiefly due to the stress and worry that the annual holiday party brings” [^29^, p. 79]. Overall, the public, media, and practitioner interest in the company Christmas party stands in sharp contrast to the dearth of empirical research on this important annual ritual in the psychological and organizational literature.

So far, the only research on the company Christmas party is a fascinating ethnographic study^10^. The author, who was a participant observation researcher at the advertising agency Shoenman and Associates in the Northeastern United States for several months, characterized the agency’s annual Christmas party as a “conspicuous social drama”:

“By 6.15 p.m. approximately 100 people had gathered in the lounge. They were clustered in small groups, some sitting around tables, others standing around the room. Drinks were free, and the bar was crowded. Before the night is over these people will have talked and drunk alcoholic beverages together, eaten dinner, scandalized their bosses and numerous other organization members in skits, drunk more, danced with each other, and otherwise socialized into the early hours of Saturday. All this is part of the annual Shoenman and Associates Christmas party, always held on the Friday evening before the holiday” (Rosen, 1988, p. 463).

In addition to the very sparse literature on the company Christmas party, two theoretical frameworks from the organizational sciences are useful for developing a better understanding of the potential antecedents of employee happiness associated with this event. First, affective events theory suggests that the interplay between individual employee characteristics (e.g., personality traits), characteristics of the work environment (e.g., job demands), and specific work events (e.g., negative feedback from a supervisor) predicts employee affective reactions, work attitudes, and behavior^18^. Numerous empirical studies have provided support for the basic tenets of affective events theory^30,31^. Based on affective events theory, the company Christmas party can be understood as a special work event and employees’ affective reactions to this event are likely predicted by various individual, work environment, and event characteristics.

Second, further support for this tripartite focus on individual, work/organizational environment, and event characteristics comes from conceptual work on fun in the workplace. In particular, this research has distinguished between person factors (e.g., person-organization fit), work characteristics (e.g., organizational role), and characteristics of fun events (e.g., activity type) which, in turn, are assumed to predict employees’ appraisals of and affective reactions to fun activities^3^. Several empirical studies have provided support for the idea that various individual, work and organizational, and event-related characteristics impact employees affective reactions to fun activities^4,5^.

I draw from various psychological theories to justify my specific hypotheses about potential relationships between employee, organizational, and event characteristics and employee happiness. First, to explain why certain employees are likely to be more happy with regard to the company Christmas party, I draw from the judgment model of subjective well-being^32^ and social identity theory^33^, which explain why certain individual characteristics and attitudes should result in greater employee happiness regarding the company Christmas party. Second, I draw from the competing values framework^23^ to justify why certain dimensions of organizational culture should be associated with greater happiness. Third, to explain why certain event characteristics should be associated with greater happiness, I draw from propositions of self-determination theory^34,35^, which suggests that event characteristics that meet existential and psychological human needs should be linked to higher happiness. Finally, I make use of psychological theorizing on rituals^1,24,36^ to argue why certain features of the company Christmas party should be associated with greater happiness. In the next section, I provide further explanations for the selection of specific potential predictors of employee happiness associated with the company Christmas party and the respective hypotheses.

## Development of hypotheses

### Employee characteristics

Based on the sparse previous research on the company Christmas party^10^ and on Christmas and organizations more generally^6,7^, I selected four individual employee characteristics as potential predictors of happiness that are conceptually closely related to this event: employees’ religion, attitude toward Christmas, organizational identification, and personal involvement in party planning (Fig. 1). First, given that Christmas is a holiday with religious roots, I expected that employees who are members of the Christian *religion* are happier with respect to the company Christmas party than employees with no or another religion. According to the judgment model of subjective well-being^32^, the extent to which the company Christmas party meets the needs of people with certain individual characteristics should result in higher happiness. Based on this model, I argue that a Christian celebration is more likely to appeal to the values and meet the spiritual needs of employees with the Christian religion. Indeed, research on person-environment fit regarding religion and spirituality suggests that a high fit is beneficial for employee happiness^37^. Second, I assumed that employees with a more favorable *attitude toward Christmas* in general are more likely to be happy with respect to the company Christmas party than employees with a less favorable attitude toward Christmas. Again, this assumption can be explain by theory and research showing that a higher fit between individual employee needs (e.g., a favorable attitude toward Christmas) and relevant workplace supplies (i.e., a Christmas party) is associated with greater happiness^38^. Third, based on research on social identity theory and health and well-being in organizational contexts^33^, I expected a positive association between *organizational identification* [i.e., the extent to which individuals perceive themselves as intertwined with the fate of their organization^39^] and employee happiness regarding the company Christmas party. Similarly, I assumed that employees’ *involvement in party planning* is associated with greater happiness regarding the event. Social identity theory suggests that employees who perceive themselves as “one” with their organization and, thus, are highly involved in organizational activities (e.g., party planning) are more likely to experience greater happiness because they feel socially included and supported^33^.

#### Hypothesis 1:

Employees with (a) Christian religion (vs. no or another religion), (b) a more favorable Christmas attitude, (c) higher organizational identification, and (d) involvement in party planning (vs. no involvement) experience greater happiness associated with the company Christmas party (i.e., higher satisfaction, lower dissatisfaction, higher high- and low-arousal positive affect, lower high- and low-arousal negative affect).

### Organizational characteristics

Given the close theoretical link between company celebrations and the construct of organizational culture (i.e., the shared assumptions, values, and beliefs of organizational members)^1^, I focus on four key dimensions of culture as organizational characteristics (Fig. 1). According to the competing values framework^23^, there are four distinct models of organizational culture that are based on combinations of three value dimensions: organizational focus (internal vs. external), structure (flexibility vs. control), and means and ends (processes vs. final outcomes). First, the *human relations* model emphasizes an internal focus, flexibility, as well as cohesion, morale, and human resource development. Second, the *internal process* model places emphasis on internal focus and control, as well information management, communication, and stability^23^. As these two models both have an inward focus combined with a human relations culture and a culture of continuity and security, respectively, I expected that employees working in organizations that endorse these cultural models are happier with respect to the company Christmas party. Company events are manifestations of an organization’s culture and, accordingly, events that represent an organizational culture associated with higher employee well-being should also lead to greater employee happiness regarding the event. Indeed, research shows that the human relations and internal process models are associated with greater positive affective outcomes among employees^40^. The third approach, the *open systems* model, stresses an external focus and flexibility, readiness, as well as growth, resource acquisition, and external support. Finally, the *rational goal* model highlights an external focus and control, goal setting and planning, as well as productivity and efficiency. Due to the outward focus of these two models and the associated cultural values of growth and efficiency, I did not expect them to be related to employees’ happiness with respect to the party.

#### Hypothesis 2:

Employees working in an organization with (a) a human relations culture and (b) an internal process culture experience greater happiness associated with the company Christmas party (i.e., higher satisfaction, lower dissatisfaction, higher high- and low-arousal positive affect, lower high- and low-arousal negative affect).

### Christmas party characteristics

I derived a number of common characteristics of company Christmas party characteristics from previous ethnographic research^10^ and media reports^27^. These characteristics can be grouped into two categories: (a) characteristics for which I could not theoretically develop clear expectations regarding their associations with employee happiness (i.e., positive, negative, or zero associations may be possible), and (b) characteristics for which directional hypotheses could be developed based on theory (see Fig. 1). The former category includes *who is invited* to the company Christmas party (i.e., only people directly associated with the company or also external guests, such as family members and friends), the *size of the party* (i.e., number of people attending), the *timing of the party* in terms of proximity to the Christmas holidays (e.g., at the beginning of December vs. a few days before the holidays), and the *location of the party* (i.e., on the organization’s premises or external). As arguments could be made for either positive or negative associations of these characteristics with employee happiness (e.g., people might enjoy smaller or larger parties more), I propose the following exploratory research question:

*Research Question 1:* How are the company Christmas party characteristics (a) invitees (only business vs. business and others), (b) party size, (c) temporal proximity to Christmas, and (d) location (outside of organizational premises vs. on organizational premises) associated with employee happiness regarding the company Christmas party (i.e., higher satisfaction, lower dissatisfaction, higher high- and low-arousal positive affect, lower high- and low-arousal negative affect)?

The second category of company Christmas party characteristics lends itself more to deductive theorizing and hypothesis development, because these characteristics are likely associated with basic human needs, including existential needs (e.g., food, beverages, safety), as well as psychological needs for competence, autonomy, and relatedness^34,35^. According to self-determination theory^34^, the fulfillment of these needs, in turn, should be related to employee happiness associated with the company Christmas party. More specifically, I expected that those employees experienced greater happiness who attended a company Christmas party that was *paid for by the organization* (rather than by themselves), and that included *fun activities*, such as games or karaoke, *music*, a *speech* (vs. no activities, no music, no speech, respectively), a la carte *food* (vs. buffet food), and the availability of both alcoholic and non-alcoholic *beverages* (vs. no choice). These event characteristics and employees engagement with them meet both existential (i.e., finances, food, drinks) and psychological needs (i.e., competence, autonomy, and relatedness can be experienced when participating in activities and listening to music and a speech) and, thus, should result in greater happiness. Moreover, I assumed that the extent of *heavy drinking* (i.e., five or more alcoholic drinks per person) and *inappropriate behavior* by either supervisors or colleagues (e.g., sexualized comments or behavior) would be negatively related to employee happiness. These assumptions are based on theory and empirical research suggesting that both heavy drinking^41,42^ and experienced incivility^43,44^ in the work context are associated with lower employee happiness.

#### Hypothesis 3:

Employees who attended a company Christmas party with (a) all costs paid by the organization (vs. by oneself), (b) fun activities (vs. no fun activities), (c) music (vs. no music), (d), a speech (vs. no speech), (e) a la carte food (vs. no or buffet food), (f) availability of both alcoholic and non-alcoholic beverages (vs. only alcoholic or non-alcoholic beverages), (g) lower levels of heavy drinking, as well as (h) no inappropriate behavior by supervisor and (i) no inappropriate behavior by colleagues experience greater happiness associated with the company Christmas party (i.e., higher satisfaction, lower dissatisfaction, higher high- and low-arousal positive affect, lower high- and low-arousal negative affect).

Finally, based on the psychological literature on rituals^1,24,36^, I expected that the greater the extent to which the company Christmas party was perceived as a ritual, the greater employees’ happiness associated with the party. In general, organizational rituals involve standardized and predictable behaviors in social situations associated with clear behavioral expectations^1^. The participation in rituals may be positively associated with happiness because they provide meaning, help manage emotions such as anxiety, and improve social connections^24,36^. To this end, a ritual experience is characterized by invariability, formality, symbolism, and the absence of clear performance expectations^45^. In the current context, *invariability* means that a company Christmas party entails a standardized and relatively inflexible pattern of behavior during the event. *Formality* involves that the company Christmas party (and employees’ behavior at the party) adheres to officially prescribed and socially accepted conventions and prescribed behavioral expectations. *Symbolism* entails that the company Christmas party is embedded with meaning and significance based on organizational members’ shared values and beliefs. Finally, the *absence of performance expectations* means that employee behavior at a company Christmas party is detached from organizational performance goals and associated expectations, and that it does not offer an immediate instrumental benefit to company productivity^24^.

#### Hypothesis 4:

Employees who attended a company Christmas party characterized by high (vs. low) (a) invariability, (b) formality, (c) symbolism, and (d) absence of performance expectations experience greater happiness associated with the party (i.e., higher satisfaction, lower dissatisfaction, higher high- and low-arousal positive affect, lower high- and low-arousal negative affect).

## Methods

### Participants and procedure

I commissioned a professional and ISO 26362 certified panel provider to recruit a sample of 800 employees from a nationally representative online panel in Germany. The survey was open during the first week of January 2019 (i.e., more than one year before the onset of the COVID-19 pandemic) and, to be eligible for inclusion, participants had to be at least 18 years old and working full-time. Of the 800 participants in the online survey, 359 reported that they recently (i.e., in December 2018) attended a company Christmas party and provided complete data for the central study variables. This final sample of participants was comprised of 210 (58.5%) men and 149 women (41.5%). Participants’ ages ranged from 19 to 67 years with a mean age of 42.81 years (*SD* = 10.62). Most participants held either a lower-secondary school degree (89; 24.8%), a higher-secondary school degree (61; 17.0%), or a college/university degree (133; 37.0%). Participants worked across 19 different industries, with the public administrative sector (13.6%), manufacturing (12.5%), various services (10.3%), and healthcare (10.0%) most represented. This heterogeneity in the organizational and occupational background of participants may enable greater generalizability of the findings (i.e., external validity). Specifically, methodologists have argued that, when true random sampling is not possible in field studies, collecting data from a heterogeneous sample of employees represents an adequate strategy to facilitate generalizability across different organizations and occupations^46,47^.

### Measures

Due to the novelty of the research topic and the non-existence of established scales for assessing certain constructs, several measures had to be developed for the purpose of this study. Before the main study, I conducted a pilot study with a convenience sample of *N = *42 employees to assess the psychometric quality of the newly developed multi-item scales (i.e., employee satisfaction and dissatisfaction with the company Christmas party, Christmas attitude, ritual characteristics). Results of the pilot study are reported below for the respective measures. In contrast, I did not pilot-test the newly developed single items included in this study, as they assess rather homogenous constructs and objective and unambiguous facts (e.g., “no” or “yes” answers regarding employees’ involvement in party planning, inappropriate behavior shown by others at the company Christmas party, fun activities organized at the party). Research has suggested that the measurement of such facts with single items can be reliable and valid^48,49^.

#### Employee happiness

Employee satisfaction and dissatisfaction with the company Christmas party were assessed with two separate scales with three self-developed items each, such as “I was satisfied with the company Christmas party” (satisfaction; Cronbach’s alpha = 0.93) and “I was disappointed with the company Christmas party” (dissatisfaction; Cronbach’s alpha = 0.91). The items were answered on 5-point response scales ranging from 1 (*strongly disagree*) to 5 (*strongly agree*). The pilot study also showed that these two scales were highly reliable (alphas > 0.80).

High- and low-arousal positive and negative affect were measured with four items each including, for instance, “enthusiastic” (high-arousal positive affect; Cronbach’s alpha = 0.92), “at ease” (low-arousal positive affect; alpha = 0.91), “nervous” (high-arousal negative affect; alpha = 0.95), and “dull” [low-arousal negative affect; alpha = 0.97^50^]. Participants were asked to indicate the extent to which they felt this way at the company Christmas party using 5-point responses scale ranging from 1 (*not at all*) to 5 (*extremely*).

#### Employee characteristics

*Religion* was assessed using multiple categories (i.e., no religion, Catholic, Lutheran, Judaism, Islam, other) and subsequently coded as 0 (*non-Christian*) or 1 (*Christian*), with the latter category including those who indicated Catholic or Lutheran. *Christmas attitude* was measured using three self-developed items, for instance “I like Christmas” (Cronbach’s alpha = 0.96; 5-point scales ranging from 1 = *strongly disagree* to 5 = *strongly agree*). The pilot study also showed that this measure was reliable (alpha = 0.90) and, according to an exploratory factor analysis with Varimax rotation, empirically distinct from employee satisfaction and dissatisfaction with the company Christmas party (i.e., the items loaded on a separate factor). *Organizational identification* was assessed with a 14-item social identification scale that was adapted to refer to “my organization,” for example “I feel a bond with my organization” [alpha = 0.97; 5-point scale ranging from 1 = strongly disagree to 5 = strongly agree^51^]. Finally, *involvement in party planning* was measured with a single item, “Did you participate in the organization of the company Christmas party?” (0 = *no*, 1 = *yes*).

#### Organizational culture

Organizational culture was assessed using 16 items from the German version of the organizational culture assessment instrument^52,53^. Example items and Cronbach’s alphas are “The organization is very a personal place. It is like an extended family. People seem to share a lot of themselves” (human relations; alpha = 0.90), “The leadership in the organization is generally considered to exemplify entrepreneurship, innovation or risk taking” (open systems; alpha = 0.87), “The management style in the organization is characterized by hard-driving competitiveness, high demands, and achievement” (rational goal; alpha = 0.83), and “The glue that holds the organization together is formal rules and policies. Maintaining a smooth-running organization is important” (internal process; alpha = 0.82). The items were answered on 5-point response scales ranging from 1 (*strongly disagree*) to 5 (*strongly agree*).

#### Christmas party characteristics

Several party characteristics were measured with single items that asked about invitees, such as organizational members, family, and friends (0 = *only business*, 1 = *business and others*), party size (i.e., estimated number of participants), temporal proximity to Christmas (i.e., number of weeks before the Christmas holidays), location (0 = *outside of organizational premises*, 1 = *on organizational premises*), costs (0 = *paid by oneself*, 1 = *paid by the organization*), fun activities, such as games or karaoke (0 = *no*, 1 = *yes*), music (0 = *no*, 1 = *yes*), speech (0 = *no*, 1 = *yes*), food (0 = *buffet*, 1 = *a la carte*), beverages (0 = *only alcoholic or non-alcoholic*, 1 = *both alcoholic or non-alcoholic*), heavy drinking (At the company Christmas party, people drank a lot of alcohol [5 or more alcoholic drinks per person], 5-point response scales ranging from 1 = *strongly disagree* to 5 = *strongly agree*), as well as inappropriate behavior by supervisor (0 = *no*, 1 = *yes*) and inappropriate behavior by colleagues (0 = *no*, 1 = *yes*).

*Ritual characteristics* were assessed with four self-developed items each. Example items and Cronbach’s alphas are “The procedure of the company Christmas party was the same as in the last years” (invariability; alpha = 0.94), “At the company Christmas party important formalities and conventions were adhered (formality; alpha = 0.79)”, “The company Christmas party represented values that are shared in my organization” (symbolism; alpha = 0.91), and “The company Christmas party had no effects on organizational performance” (absence of performance expectations; alpha = 0.86). The items were answered on 5-point response scales ranging from 1 (*strongly disagree*) to 5 (*strongly agree*). The pilot study also showed that these four scales were highly reliable (alphas > 0.78) and, according to an exploratory factor analysis with Varimax rotation, empirically distinct from each other (i.e., the 16 items had their highest loadings on four separate factors, with loadings ranging in size from 0.58 to 0.91).

#### Control variables

I controlled for a number of employee characteristics that have been shown to be associated with general happiness^54–56^, including age (measured in years since birth), sex (1 = *male*, 2 = *female*), educational level (1 = *some high school*, 7 = *university degree*), organizational tenure (in years), and job level (1 = *entry level*, 7 = *executive level*).

### Ethics approval

According to national regulations by the German Science Foundation (see https://www.dfg.de/en/research_funding/faq/faq_humanities_social_science/index.html, “When do I need a statement by an ethics committee?”, section “II. Information on proposals from the field of psychology”), a statement by an ethics committee on a psychological study is only required “if the participants are expected to take risks, if the study involves a high level of (physical or emotional) stress and/or if the participants are not to be fully informed as to the aims and procedures of the study; where studies involve patients; if fMRI and electrical or magnetic stimulation (e.g. TMS) are to be used; in the case of psychopharmacological studies.” None of these criteria applied to the current correlational survey study and, hence, no ethical approval statement was obtained from an ethics committee. All procedures performed in this study, which involved human participants, were in accordance with the ethical standards of the institutional and/or national research committee and with the 1964 Helsinki Declaration and its later amendments or comparable ethical standards.

### Informed consent

Informed consent about participation and publication was obtained from all individual participants included in the study. Participation in the study was voluntary, and data were saved and processed anonymously.

## Results

Descriptive statistics and correlations among the study variables are shown in Table 1. A number of interesting bivariate associations emerged between employee happiness regarding the company Christmas party and employee characteristics. For instance, older and longer tenured employees were less dissatisfied and experienced less negative affect with respect to the party, and employees with higher job level, organizational identification, Christmas attitude, and planning involvement tended to be happier with respect to the party. Moreover, higher scores on all organizational culture characteristics were generally associated with greater happiness regarding the company Christmas party. Finally, party characteristics were differentially correlated with specific indicators employee happiness. Interestingly, a la carte food, availability of alcoholic and non-alcoholic beverages, and symbolism were most consistently associated with higher satisfaction, lower dissatisfaction, and lower negative affect (see Table 1).

**Table 1:** Descriptive statistics and correlations.

Variables	*M*	*SD*	1	2	3	4	5	6	7	8	9	10	11	12	13	14	15	16	17
**Employee characteristics**																			
1. Age	42.81	10.62	–																
2. Sex^a^	1.42	0.49	− 0.02	–															
3. Educational level	5.41	1.55	− 0.19**	− 0.02	–														
4. Religion^b^	0.49	0.40	− 0.04	− 0.03	0.06	–													
5. Organizational tenure	10.89	9.89	0.51**	− 0.12*	− 0.10	0.05	–												
6. Job level	4.04	1.35	0.12*	− 0.09	0.19**	0.06	0.25**	–											
7. Christmas attitude	3.77	1.13	− 0.04	− 0.01	0.02	0.15**	0.00	0.03	(0.96)										
8. Organizational identification	3.49	0.84	0.11*	− 0.01	− 0.09	0.01	0.02	0.22**	0.24**	(0.97)									
9. Planning involvement^c^	0.20	0.40	− 0.01	0.04	0.07	0.07	− 0.01	0.24**	0.08	0.12*	–								
**Organizational culture**																			
10. Human relations	3.48	0.90	0.07	0.01	− 0.03	0.01	− 0.05	0.23**	0.17**	0.78**	0.12*	(0.90)							
11. Open systems	3.34	0.93	0.02	− 0.04	− 0.02	− 04	− 0.05	0.23**	0.18**	0.70**	0.06	0.78**	(0.87)						
12. Rational goal	3.43	0.86	0.02	− 0.04	− 0.08	− 0.09	− 0.06	0.16**	0.19**	0.56**	0.04	0.59**	0.81**	(0.83)					
13. Internal process	3.61	0.76	0.13*	− 0.00	− 0.12*	− 0.06	0.05	0.17**	0.17**	0.71**	0.11*	0.76**	0.67**	0.64**	(0.82)				
**Christmas party characteristics**																			
14. Invitees^d^	0.10	0.30	0.01	− 0.05	− 0.02	0.13*	0.03	0.16**	0.10	0.16**	0.19**	0.15**	0.16**	0.14**	0.14**	–			
15. Party size	100.72	178.31	− 0.05	− 0.02	0.13**	0.07	− 0.02	0.06	0.09	0.09	0.01	0.07	0.19**	0.21**	0.07	0.13*	–		
16. Proximity to Christmas	2.67	1.00	− 0.03	− 0.08	− 0.01	0.02	− 0.02	0.02	0.07	0.00	0.04	− 0.02	− 0.00	− 0.08	− 0.07	0.00	− 0.00	–	
17. Location^e^	0.28	0.45	0.04	− 0.09	− 0.06	0.02	0.09	− 0.00	− 0.04	− 0.03	0.10	− 0.06	− 0.05	− 0.09	− 0.03	0.01	0.10	0.13*	–
18. Costs^f^	0.87	0.33	0.12*	0.00	− 0.05	− 0.02	− 0.03	0.03	0.02	− 0.01	− 0.06	− 0.03	0.01	0.05	− 0.08	− 0.01	0.12*	0.07	− 0.01
19. Activities^g^	0.49	0.50	− 0.08	0.00	0.02	0.00	− 0.09	0.09	0.05	0.06	0.12*	0.10	0.11*	0.05	0.09	0.09	0.08	0.01	− 0.03
20. Music^h^	0.19	0.39	− 0.08	0.06	− 0.08	0.03	− 0.04	− 0.05	− 0.02	0.03	0.01	0.09	0.02	0.02	0.05	0.03	0.04	0.05	− 0.08
21. Speech^i^	0.55	0.50	0.02	0.02	0.01	− 0.00	− 0.08	− 0.05	− 0.07	− 0.04	0.00	0.00	0.02	0.01	− 0.04	− 0.06	0.16*	0.03	0.06
22. Food^j^	0.37	0.48	0.08	0.07	− 0.09	− 0.07	0.06	0.00	− 0.04	0.05	− 0.08	0.03	− 0.04	0.04	0.07	− 0.02	− 0.24**	− 0.09	− 0.42**
23. Beverages^k^	0.91	0.29	0.04	− 0.03	− 0.09	− 0.12*	− 0.03	− 0.11	0.01	0.02	− 0.14**	0.08	0.07	0.10	0.06	− 0.10	− 0.06	− 0.09	− 0.22**
24. Heavy drinking	2.72	1.26	− 0.15**	− 0.12*	− 0.11*	− 0.05	− 0.07	0.00	0.06	0.06	− 0.03	0.12*	0.22**	0.25**	0.11*	0.04	0.25**	− 0.02	− 0.09
25. Inappropriate beh. superv.^l^	0.17	0.37	− 0.15**	0.00	− 0.01	− 0.01	− 0.15**	0.01	− 0.03	− 0.07	0.00	− 0.03	0.04	0.06	− 0.05	0.00	0.09	0.03	− 0.04
26. Inappropriate beh. colleag.^m^	0.03	0.18	− 0.14**	− 0.03	− 0.01	0.10	− 0.12*	0.04	− 0.00	− 0.04	0.10	− 0.00	0.06	0.01	− 0.04	0.04	0.07	0.05	− 0.05
27. Invariability	3.50	1.11	− 0.04	0.03	− 0.05	0.07	0.00	0.01	0.05	0.0.13*	− 0.08	0.12*	0.20**	0.23**	0.12*	0.06	0.14**	0.07	0.05
28. Formality	3.65	0.84	0.10*	− 0.03	− 0.04	0.04	0.13*	0.12*	0.11*	0.26**	− 0.04	0.20**	0.17**	0.17**	0.18**	0.08	0.05	− 0.01	0.03
29. Symbology	3.68	0.97	0.06	0.00	− 0.09	0.02	− 0.03	0.19**	0.21**	0.61**	0.07	0.64**	0.53**	0.44**	0.53**	0.13*	0.16**	0.03	− 0.05
30. Absence of perf. expect.^n^	3.61	0.98	0.01	0.07	− 0.06	0.08	0.02	− 0.05	− 0.01	0.05	− 0.05	− 0.02	− 0.03	.02	0.02	− 0.03	0.06	0.03	− 0.02
**Employee happiness**																			
31. Satisfaction with party	4.08	0.88	0.06	0.10	− 0.06	0.01	0.02	0.12	0.19**	0.50**	0.12*	0.55**	0.40**	0.34**	0.48**	0.09	0.07	− 0.01	− 0.15**
32. Dissatisfaction with party	1.73	1.03	− 0.20**	− 0.07	0.06	0.03	− 0.13*	− 0.03	− 0.06	− 0.28**	− 0.02	− 0.37**	− 0.22**	− 0.16**	− 0.33**	− 0.01	0.08	0.04	0.09
33. High-arousal positive affect	3.29	1.04	− 0.10	− 0.01	− 0.07	0.04	− 0.08	0.18**	0.23**	0.61**	0.20**	0.59**	0.53**	0.43**	0.48**	0.15**	0.13*	0.01	− 0.09
34. Low-arousal positive affect	3.79	0.87	0.15**	− 0.02	− 0.04	− 0.07	0.04	0.17**	0.14**	0.49**	0.09	0.48**	0.37**	0.31**	0.52**	0.15**	0.00	0.02	− 0.06
35. High-arousal negative affect	1.37	0.75	− 0.24**	− 0.04	0.07	0.08	− 0.13*	0.02	− 0.02	− 0.11*	0.07	− 0.15**	− 0.02	− 0.02	− 0.15**	− 0.02	0.08	0.04	0.04
36. Low-arousal negative affect	1.42	0.83	− 0.22**	− 0.09	0.06	0.06	− 0.11*	0.00	− 0.05	− 0.20**	0.02	− 0.21**	− 0.08	− 0.04	− 0.20**	− 0.02	0.06	0.07	0.03

Variables	18	19	20	21	22	23	24	25	26	27	28	29	30	31	32	33	34	35	36
18. Costs^f^	–																		
19. Activities^g^	-0.03	–																	
20. Music^h^	− 0.03	0.03	–																
21. Speech^i^	0.20**	0.13*	− 0.01	–															
22. Food^j^	− 0.10	− 0.10	− 0.02	− 0.23**	–														
23. Beverages^k^	− 0.03	− 0.01	− 0.02	0.03	0.12*	–													
24. Heavy drinking	0.09	0.07	0.11*	0.06	− 0.07	0.19**	–												
25. Inappropriate beh. superv.^l^	0.13*	0.19**	0.01	0.08	− 0.10	− 0.04	0.35**	–											
26. Inappropriate beh. colleag.^m^	− 0.02	0.07	0.07	− 0.08	− 0.11*	− 0.27**	0.17**	0.42**	–										
27. Invariability	0.09	− 0.10	− 0.03	− 0.04	0.03	− 0.03	0.14**	0.00	0.04	(0.94)									
28. Formality	0.08	0.03	0.03	0.04	− 0.02	− 0.09	− 0.09	− 0.21**	− 0.02	0.34**	(0.79)								
29. Symbolism	0.09	0.12*	0.09	0.07	0.06	0.00	0.17**	− 0.05	− 0.04	0.19**	0.36**	(0.91)							
30. Absence of perf. expect.^n^	0.10	− 0.03	0.01	− 0.04	0.07	0.00	0.12*	− 0.04	0.01	0.22**	0.04	− 0.03	(0.86)						
**Employee happiness**																			
31. Satisfaction with party	− 0.02	0.15**	0.06	0.03	0.14**	0.09	0.06	− 0.08	− 0.10	0.03	0.10	0.64**	0.01	(0.93)					
32. Dissatisfaction with party	0.05	− 0.03	0.05	− 0.13*	− 0.15**	− 0.21**	0.10	0.11*	0.22**	0.08	0.08	− 0.37**	0.11*	− 0.67**	(0.91)				
33. High-arousal positive affect	− 0.06	0.15**	0.07	− 0.01	0.05	− 0.01	0.17**	− 0.05	0.05	0.08	0.09	0.62**	− 0.02	0.73**	− 0.41**	(0.92)			
34. Low-arousal positive affect	− 0.03	0.09	− 0.01	− 0.06	0.10	0.01	0.03	− 0.09	− 0.03	0.11*	0.19**	0.43**	0.07	0.58**	− 0.35**	0.57**	(0.91)		
35. High-arousal negative affect	0.03	− 0.00	0.04	− 0.10	− 0.15**	− 0.19**	0.19**	0.14**	0.28**	0.09	0.01	− 0.10	0.09	− 0.29**	0.62**	− 0.02	− 0.22**	(0.95)	
36. Low-arousal negative affect	0.04	− 0.04	0.01	− 0.06	− 0.12*	− 0.11*	0.13*	0.06	0.24**	0.13*	0.05	− 0.19**	0.11*	− 0.35**	0.62**	− 0.12*	− 0.23**	0.82**	(0.97)

N = 359. ^a^Sex (1 = male, 2 = female), ^b^Religion (0 = non-Christian, 1 = Christian), ^c^Planning involvement (0 = no, 1 = yes), ^d^Invitees (0 = only business, 1 = business and others), ^e^Location (0 = outside of organizational premises, 1 = on organizational premises), ^f^Costs (0 = paid by oneself, 1 = paid by organization), ^g^Activities (0 = no, 1 = yes), ^h^Music (0 = no, 1 = yes), ^i^Speech (0 = no, 1 = yes), ^j^Food (0 = buffet, 1 = a la carte), ^k^Beverages (0 = only alcoholic or non-alcoholic, 1 = both alcoholic and non-alcoholic), ^l^Inappropriate behavior by supervisor (0 = no, 1 = yes), ^m^Inappropriate behavior by colleagues (0 = no, 1 = yes), ^n^Absence of performance expectations. Reliability estimates (Cronbach's alpha), where available, are provided on the diagonal.

### Results of regression analyses

Table 2 shows that the results of multiple regression analyses used to test the hypotheses. According to Hypothesis 1, employees with (a) Christian religion, (b) a more favorable Christmas attitude, (c) higher organizational identification, and (d) involvement in party planning experience greater happiness regarding the company Christmas party. As shown in Table 2, religion and Christmas attitude did not emerge as significant predictors of happiness in the analyses and, therefore Hypotheses 1a and 1b were not supported. Organizational identification (*β* = 0.28, *p* < 0.01) and planning involvement (*β* = 0.10, *p* < 0.05) only predicted high-arousal positive affect, but not the other happiness outcomes, in the expected direction. Thus, Hypotheses 1c and 1d were only partially supported.

**Table 2:** Results of regression analyses predicting employee happiness outcomes.

Predictors	Satisfaction with party			Dissatisfaction with party			High-arousal positive affect			Low-arousal positive affect			High-arousal negative affect			Low-arousal negative affect		
	*B*	*SE*	*β*	*B*	*SE*	*β*	*B*	*SE*	*β*	*B*	*SE*	*β*	*B*	*SE*	*β*	*B*	*SE*	*β*
**Employee characteristics**																		
Age	− 0.00	0.00	− 0.04	− 0.01	0.01	− 0.06	− **0.01**	**0.00**	− 0.14**	0.01	0.01	0.10	− **0.01**	**0.00**	− 0.14*	− **0.01**	**0.01**	− 0.14*
Sex^a^	0.14	0.07	0.08	− 0.11	0.09	− 0.05	− 0.02	0.08	− 0.01	− 0.04	0.08	− 0.02	− 0.02	0.08	− 0.01	− 0.11	0.09	− 0.06
Educational level	− 0.00	0.02	− 0.01	− 0.01	0.03	− 0.02	− 0.04	0.03	− 0.06	0.03	0.03	0.05	0.01	0.03	0.02	− 0.00	0.03	− 0.00
Religion^b^	− 0.02	0.07	− 0.01	0.01	0.09	0.01	0.01	0.08	0.01	− 0.16	0.08	− 0.09	0.09	0.08	0.06	0.05	0.09	0.03
Organizational tenure	0.01	0.00	0.09	− **0.02**	**0.01**	− 0.16**	0.00	0.01	0.01	− 0.00	0.01	− 0.04	− 0.00	0.01	− 0.05	− 0.01	0.01	− 0.07
Job level	− 0.02	0.03	− 0.03	0.06	0.04	0.07	0.02	0.03	0.03	0.03	0.03	0.04	0.02	0.03	0.04	0.03	0.04	0.05
Christmas attitude	0.04	0.02	0.05	− 0.01	0.04	− 0.01	0.05	0.04	0.06	0.02	0.04	0.03	− 0.03	0.03	− 0.04	− 0.03	0.04	− 0.04
Org. identification	0.06	0.07	0.06	0.06	0.10	0.05	**0.34**	**0.08**	0.28**	0.15	0.08	0.15	0.02	0.08	0.03	− 0.08	0.09	− 0.08
Planning involvement^c^	0.11	0.09	0.05	0.02	0.12	0.01	**0.25**	**0.10**	0.10*	0.02	0.11	0.01	0.14	0.10	0.07	0.09	0.11	0.05
**Organizational culture**																		
Human relations	**0.24**	**0.08**	0.25**	− **0.28**	**0.11**	− 0.25*	0.15	0.09	0.13	0.05	0.10	0.05	− **0.22**	**0.09**	− 0.26*	− 0.14	0.10	0.05
Open systems	− 0.16	0.09	− 0.17	0.08	0.11	0.07	0.02	0.10	0.02	− 0.06	0.10	− 0.06	0.14	0.09	0.17	0.06	0.10	0.07
Rational goal	0.07	0.08	0.06	0.05	0.10	0.04	0.06	0.08	0.05	− 0.08	0.09	− 0.08	0.01	0.08	0.01	0.09	0.09	0.09
Internal process	0.06	0.08	0.05	− 0.15	0.10	− 0.11	− 0.09	0.09	− 0.06	**0.38**	**0.09**	0.33**	− 0.05	0.08	− 0.05	− 0.07	0.09	− 0.06
**Party characteristics**																		
Invitees^d^	− 0.03	0.12	− 0.01	− 0.01	0.16	− 0.00	− 0.00	0.14	0.00	0.20	0.14	0.07	− 0.14	0.13	− 0.06	− 0.07	0.14	− 0.02
Party size	0.00	0.00	0.02	0.00	0.00	0.05	0.00	0.00	0.02	0.00	0.00	− 0.04	0.00	0.00	− 0.02	0.00	0.00	− 0.01
Proximity to Christmas	0.02	0.04	0.02	0.00	0.05	0.00	− 0.01	0.04	− 0.01	0.03	0.04	0.04	0.00	0.04	0.00	0.04	0.04	0.04
Location^e^	− **0.19**	**0.09**	− 0.10*	0.09	0.12	0.03	− 0.13	0.10	− 0.06	− 0.06	0.10	− 0.03	− 0.02	0.10	− 0.01	− 0.04	0.11	− 0.02
Costs^f^	− 0.09	0.11	− 0.03	0.12	0.14	0.04	− 0.17	0.12	− 0.06	− 0.05	0.13	− 0.02	0.08	0.12	0.03	0.10	0.13	0.04
Activities^g^	**0.14**	**0.07**	0.08*	− 0.00	0.09	0.00	0.14	0.08	0.07	0.08	0.08	0.05	− 0.03	0.08	− 0.02	− 0.03	0.09	− 0.02
Music^h^	− 0.03	0.09	− 0.01	0.16	0.12	0.06	− 0.03	0.10	− 0.01	− 0.10	0.10	− 0.04	0.04	0.10	0.02	− 0.00	0.11	− 0.00
Speech^i^	0.03	0.07	0.02	− **0.31**	**0.10**	− 0.15**	0.01	0.08	0.01	− 0.06	0.08	− 0.04	− **0.18**	**0.08**	− 0.12*	− 0.10	0.09	− 0.06
Food^j^	0.07	0.08	0.04	− 0.12	0.11	− 0.06	0.03	0.10	0.01	0.04	0.10	0.02	− 0.16	0.09	− 0.10	− 0.12	0.10	− 0.07
Beverages^k^	0.12	0.14	0.04	− **0.53**	**0.18**	− 0.15**	− 0.12	0.15	− 0.03	0.01	0.16	0.00	− **0.33**	**0.15**	− 0.12*	− 0.16	0.16	− 0.05
Heavy drinking	− 0.04	0.03	− 0.05	**0.14**	**0.04**	0.17**	00.04	0.04	0.05	0.00	0.04	0.00	**0.11**	**0.04**	0.18**	**0.09**	**0.04**	0.13*
Inapp. beh. superv.^l^	− 0.09	0.11	− 0.04	− 0.02	0.15	− 0.01	− **0.32**	**0.13**	− 0.12*	− 0.11	0.13	− 0.05	− 0.05	0.12	− 0.02	− 0.23	0.13	− 0.11
Inapp. beh. colleag.^m^	− 0.18	0.23	− 0.04	0.46	0.30	0.08	0.34	0.25	0.06	0.15	0.26	0.03	**0.64**	**0.24**	0.15**	**0.81**	**0.27**	0.18**
Invariability	− 0.03	0.04	− 0.04	0.01	0.05	0.01	− 0.00	0.04	− 0.00	0.03	0.03	0.03	0.02	0.04	0.03	0.06	0.04	0.08
Formality	− **0.15**	**0.05**	− 0.14**	**0.32**	**0.06**	0.26**	− **0.20**	**0.05**	− 0.16**	0.02	0.06	0.02	0.07	0.05	0.07	**0.13**	**0.06**	0.13*
Symbolism	**0.48**	**0.05**	0.52**	− **0.40**	**0.07**	− 0.38**	**0.42**	**0.06**	0.39**	**0.16**	**0.06**	0.17**	− 0.03	0.06	− 0.04	− **0.14**	**0.06**	− 0.17*
Absence of performance expectations	0.04	0.04	0.04	0.06	0.05	0.06	− 0.01	0.04	− 0.01	0.06	0.04	0.07	0.04	0.04	0.05	0.06	0.04	0.07
R^2^		0.53			0.40			0.57			0.37			0.23			0.23	
F	12.15**			7.34**			14.22**			6.31**			3.34**			3.27**		

N = 359. Significant effects are highlighted in bold font. ^a^Sex (1 = male, 2 = female), ^b^Religion (0 = non-Christian, 1 = Christian), ^c^Planning involvement (0 = no, 1 = yes), ^d^Invitees (0 = only business, 1 = business and others), ^e^Location (0 = outside of organizational premises, 1 = on organizational premises), ^f^Costs (0 = paid by oneself, 1 = paid by organization), ^g^Activities (0 = no, 1 = yes), ^h^Music (0 = no, 1 = yes), ^i^Speech (0 = no, 1 = yes), ^j^Food (0 = buffet, 1 = a la carte), ^k^Beverages (0 = only alcoholic or non-alcoholic, 1 = both alcoholic and non-alcoholic), ^l^Inappropriate behavior by supervisor (0 = no, 1 = yes), ^m^Inappropriate behavior by colleagues (0 = no, 1 = yes).

*p < 0.05, **p < 0.01.

Hypothesis 2 states that employees working in a company with (a) a human relations culture and (b) an internal process culture experience higher happiness regarding the company Christmas party. These hypotheses were also only partially supported, as human relations culture only positively predicted satisfaction (*β* = 0.25, *p* < 0.01) and negatively predicted both dissatisfaction (*β* = − 0.25, *p* < 0.05) and high-arousal negative affect (*β* = − 0.26, *p* < 0.05), and internal process culture only positively predicted low-arousal positive affect (*β* = 0.33, *p* < 0.01). Open systems and rational goal cultures did not significantly predict happiness outcomes (see Table 2).

Regarding Research Question 1, results showed that invitees, party size, and temporal proximity to Christmas were not significantly associated with employee happiness regarding the company Christmas party. Moreover, location only predicted satisfaction (*β* = − 0.10, *p* < 0.05), suggesting that employees who attended a party on organizational premises were somewhat less satisfied than employees who attended a party outside of organizational premises.

Hypothesis 3 received mixed support, as only some party characteristics differentially predicted happiness outcomes in the expected directions (see Table 2). Costs, music, and food did not significantly predict any outcomes. Thus, Hypotheses 3a, 3c, and 3e were not supported. In contrast, fun activities positively predicted satisfaction (*β* = 0.08, *p* < 0.05), and a speech negatively predicted dissatisfaction (*β* = − 0.15, *p* < 0.01) and high-arousal negative affect (*β* = − 0.12, *p* < 0.05). Similarly, availability of both alcoholic and non-alcoholic beverages negatively predicted dissatisfaction (*β* = − 0.15, *p* < 0.01) and high-arousal negative affect (*β* = − 0.12, *p* < 0.05). Also as expected, heavy drinking positively predicted dissatisfaction (*β* = 0.17, *p* < 0.01), high-arousal negative affect (*β* = 0.18, *p* < 0.01), and low-arousal negative affect (*β* = 0.13, *p* < 0.05). Inappropriate supervisor behavior negatively predicted high-arousal positive affect (*β* = − 0.12, *p* < 0.05), whereas inappropriate colleague behavior positively predicted both high-arousal negative affect (*β* = 0.15, *p* < 0.01) and low arousal negative affect (*β* = 0.18, *p* < 0.01). Overall, therefore, Hypotheses 3b, 3d, 3f, 3g, 3h, and 3i received mixed support.

Finally, Hypothesis 4 suggested that a company Christmas party characterized by high (a) invariability, (b) formality, (c) symbolism, and (d) absence of performance expectations is associated with higher happiness. Hypothesis 4c was largely supported by the results, as symbolism positively predicted satisfaction (*β* = 0.52, *p* < 0.01), high-arousal positive affect (*β* = 0.39, *p* < 0.01), and low-arousal positive affect (*β* = 0.17, *p* < 0.01), and negatively predicted dissatisfaction (*β* = − 0.38, *p* < 0.01) and low-arousal (but not high-arousal) negative affect (*β* = − 0.17, *p* < 0.05). Unexpectedly, and in contrast to Hypothesis 4b, formality negatively predicted satisfaction (*β* = − 0.14, *p* < 0.01) and high-arousal positive affect (*β* = − 0.16, *p* < 0.01), and positively predicted dissatisfaction (*β* = 0.26, *p* < 0.01) and low-arousal negative affect (*β* = 0.13, *p* < 0.05). Invariability and absence of performance expectations did not have significant effects on happiness outcomes and, thus, Hypotheses 4a and 4d were also not supported.

## Discussion

The goal of this study was to explore relationships of various individual, organizational, and event characteristics with employees’ happiness associated with a recent company Christmas party they attended. In summary, results showed that a human relations culture, an external location, fun activities, informality, and symbolism predicted higher employee satisfaction with the Christmas party. In contrast, heavy drinking and formality predicted higher dissatisfaction with the Christmas party, whereas organizational tenure, a human relations culture, a speech, providing both alcoholic and non-alcoholic beverages, and symbolism predicted lower dissatisfaction. Furthermore, several characteristics, including employee age, organizational identification, involvement in planning, a human relations and an internal process culture, a speech, providing both alcoholic and non-alcoholic beverages, heavy drinking, supervisor and coworker inappropriate behavior, formality, and symbolism, were differentially associated with high- and low-arousal positive and negative affect.

### Theoretical and practical implications

This quantitative study draws from and extends early qualitative research on the company Christmas party, which was based on ethnographic observation in an advertising agency^10^. Drawing from affective events theory^18^ and conceptual research on fun in the workplace^3^, I distinguished potential antecedents of employee happiness associated with the company Christmas party into employee, organizational, and event-related characteristics. Accordingly, this study contributes to these theoretical frameworks by conceptualizing the company Christmas party—a relatively rare (i.e., annual), but particularly fun and meaningful event for many employees—as an affective event and fun activity in the workplace. Based on theories of human needs and well-being^32^, person-environment fit^37^, and social identification^39^, I developed hypotheses on links between employee characteristics and happiness regarding the company Christmas party. Results showed that age, organizational tenure, organizational identification, and planning involvement emerged as predictors of select happiness outcomes. These findings are consistent with the lifespan literature, which suggests that older adults possess better emotion regulation skills than younger adults and, thus, experience higher subjective well-being^57^. Moreover, the findings on organizational identification and planning involvement are consistent with research suggesting that a close bond and involvement with one’s organization benefits well-being^33^. Future theory development and empirical research could, therefore, focus on the role of age and organizational identification and involvement for employee happiness in the context of organizational events.

Based on the well-established competing values theoretical framework^23^, I considered links between different models of organizational culture and employee happiness regarding the company Christmas party. Consistent with expectations, the human relations model and the internal process model positively predicted certain happiness outcomes. Thus, future theorizing and research on important organizational events should consider the role of these models of organizational culture in addition to employee and event characteristics.

Results for the party characteristics were very mixed, but provided some support for expectations based on theories on basic human needs, such as safety and stimulation^35^, and the psychology of rituals^24,36^. Interestingly, certain party characteristics seemed to play a particularly important role in terms of lower dissatisfaction and negative affect (e.g., speech, variety of alcoholic and non-alcoholic beverages), or in terms of higher dissatisfaction and negative affect (e.g., heaving drinking, inappropriate colleague behavior). Future theory building and empirical research could focus on the reasons for these differential effects on positive and negative indicators of employee happiness.

In terms of practical implications, the current findings may offer some preliminary practical suggestions to organizations and committees tasked with organizing a company Christmas party. To make the party a success, organizational leaders could generally attempt to establish a human relations culture that focuses on flexibility, cohesion, morale, and employee development, as well as an internal process culture that provides continuity and safety^23^. Furthermore, they could find ways to enhance employees’ organizational identification and involvement in planning the party (e.g., by assigning various roles based on employees’ abilities and needs), even if it takes place outside of the organization’s premises. In terms of ritual features, the party should clearly represent the values of the organization while not being overly formal at the same time. Even more practically, the party should include fun activities, a speech by a manager, and both alcoholic and non-alcoholic beverages to enhance happiness. Finally, heavy drinking and inappropriate behaviors by supervisors and colleagues should be prevented, for instance by clearly communicating rules and norms for appropriate behavior before the party, and by punishing employees who do not stick to the rules^28,29^.

## Limitations and future research

This study has a number of limitations that could be addressed by future research on the company Christmas party or other important events. First, the cross-sectional research design and correlational analyses do not allow conclusions about causality. Future studies should adopt longitudinal or quasi-experimental designs and path analysis to draw more definite inferences.

Second, the convenience sample with data collected from employees working in various organizations and occupations may raise concerns about the generalizability of findings (i.e., external validity). Indeed, employee happiness may differ across organizations and occupations^58^. However, it has been argued that collecting data from a heterogeneous sample of employees represents an adequate strategy to facilitate generalizability across different organizations and occupations, especially when true random sampling is not possible in field studies^46,47^.

Third, all data were self-reported by employees and, therefore, common method bias might be a concern (i.e., artificially inflated correlations due to all responses coming from a single source). To address this potential problem, and consistent with recommendations by methodologists^59^, I used different item response formats in the survey to measure the various predictor and outcome variables in this study (e.g., rating scales, yes/no checklists). Results suggested a differential pattern of effects, including many non-significant associations, suggesting that common method bias may not be a significant concern in this study.

Fourth, the use of single-item measures may be criticized for not capturing complex constructs well (i.e., potentially low content validity), for fewer points of discrimination (i.e., lower sensitivity), and for lacking an estimate of internal-consistency reliability^60^. However, research has suggested that the measurement of rather homogenous constructs and objective and unambiguous facts (e.g., “no” or “yes” answers regarding employees’ involvement in party planning) with single items can be reliable and valid^48,49^. Nevertheless, future research should attempt to use established multi-items scales and to obtain data from multiple sources, including archival data as well as survey data obtained from employees, leaders, coworkers, and other people who attended the Christmas party.

Fifth, a related issue is that responses to subjective well-being measures, such as satisfaction and dissatisfaction, tend to be positively and negatively skewed, respectively^61^. The results of this study are not exception (see descriptive statistics in Table 1). However, the relatively large sample size in the current study renders the results of regression analyses rather robust against deviations from the normality assumption. Moreover, given potential problems regarding the interpretation of results, we decided to not statistically transform the outcome measures^62^.

Sixth, given the dearth of previous theorizing and empirical research on the company Christmas party, I drew from a number of different theoretical approaches and included a large variety of predictors. Based on the current findings, future research could focus on a narrower set of potential predictors and additionally explore possible mechanisms (i.e., mediators) and boundary conditions (i.e., moderators) of their effects.

Finally, the satisfaction and affect outcomes in this study were conceptualized and measured as context-specific indicators (i.e., experiences associated with the company Christmas party) of employees’ subjective well-being or happiness. Future studies could examine how these context-specific outcomes, as well as the various predictors considered in this study, relate to broader and de-contextualized indicators of happiness, such as life satisfaction and general positive and negative affect^12,13^.

## Conclusion

The company Christmas party constitutes an important annual event that is more or less eagerly anticipated by employees. This study represents a first attempt to shed light on potential predictors of employee happiness associated with the Christmas party at the individual, organizational, and event levels. The differential findings may help researchers select theories and variables for use in future studies, and they provide practitioners with some preliminary ideas on how the Christmas party could be designed to maximize employee happiness.

## Data Availability

The dataset used in the current study is available from the corresponding author on reasonable request.
